# Fingolimod Plays Role in Attenuation of Myocardial Injury Related to Experimental Model of Cardiac Arrest and Extracorporeal Life Support Resuscitation

**DOI:** 10.3390/ijms20246237

**Published:** 2019-12-11

**Authors:** Naseer Ahmed, Abid H. Laghari, Bashar AlBkhoor, Sobia Tabassum, Sultan Ayoub Meo, Nazeer Muhammad, Daniele Linardi, Abeer A. Al-Masri, Guido Fumagalli, Giovanni Battista Luciani, Giuseppe Faggian, Alessio Rungatscher

**Affiliations:** 1 Department of Biological and Biomedical Sciences, The Aga Khan University, Karachi 74800, Pakistan; 2 Department of Surgery, Cardiac Surgery Division, University of Verona Medical School, 37129 Verona, Italy; daniele.linardi@univr.it (D.L.); giovanni.luciani@univr.it (G.B.L.); giuseppe.faggian@univr.it (G.F.); alessio.rungatscher@univr.it (A.R.); 3 Department of Medicine, section of Cardiology, Aga Khan University, Karachi 74800, Pakistan; abid.laghari@aku.edu; 4 Queen Ali Heart Institute, Amman 111881, Jordan; albkhoorbashar@gmail.com; 5 Department of Biological Sciences, International Islamic University, Islamabad 44000, Pakistan; sobia.tabasum@iiu.edu.pk; 6 Department of Physiology, College of Medicine, King Saud University, Riyadh 11461, Saudi Arabia; smeo@ksu.edu.sa (S.A.M.); aelmasri@ksu.edu.sa (A.A.A.-M.); 7 COMSATS University Islamabad, Wah Campus, Rawalpindi 47040, Pakistan; nazeermuhammad@ciitwah.edu.pk; 8 Department of Diagnostics and Public Health, Section of Pharmacology, University of Verona Medical School, 37134 Verona, Italy; guido.fumagalli50@gmail.com

**Keywords:** fingolimod, cardioprotection, cardiac arrest, extracorporeal life support, resuscitation

## Abstract

Background: Sudden cardiac arrest is a major global health concern, and survival of patients with ischemia–reperfusion injury is a leading cause of myocardial dysfunction. The mechanism of this phenomenon is not well understood because of the complex pathophysiological nature of the disease. Aim of the study was to investigate the cardioprotective role of fingolimod in an in vivo model of cardiac arrest and resuscitation. Methods: In this study, an in vivo rat model of cardiac arrest using extracorporeal membrane oxygenation resuscitation monitored by invasive hemodynamic measurement was developed. At the beginning of extracorporeal life support (ECLS), animals were randomly treated with fingolimod (Group A, *n* = 30) or saline (Group B, *n* = 30). Half of the animals in each group (Group A1 and B1, *n* = 15 each) were sacrificed after 1 h, and the remaining animals (Group A2 and B2) after 24 h of reperfusion. Blood and myocardial tissues were collected for analysis of cardiac features, inflammatory biomarkers, and cell signaling pathways. Results: Treatment with fingolimod resulted in activation of survival pathways resulting into reduced inflammation, myocardial oxidative stress and apoptosis of cardiomyocytes. This led to significant improvement in systolic and diastolic functions of the left ventricle and improved contractility index. Conclusions: Sphingosine1phosphate receptor activation with fingolimod improved cardiac function after cardiac arrest supported with ECLS. Present study findings strongly support a cardioprotective role of fingolimod through sphingosine-1-phosphate receptor activation during reperfusion after circulatory arrest.

## 1. Introduction

Despite rapid advancements in the field of medicine worldwide, sudden cardiac arrest is still a common cause of death in patients with hereditary and ischemic heart diseases [1,2]. The post-resuscitation mortality rate in such patients is about 50% [3]. Both cardiac arrests and cardiopulmonary resuscitation are associated with global myocardial ischemia–reperfusion injury which leads to myocardial dysfunction, resulting in poor prognosis and adverse outcomes [4]. Currently, extracorporeal life support (ECLS) is an effective way of treating cardiogenic shock or cardiac arrest because of its great potential to provide swift circulatory support via peripheral vascular access [5]. Sphingosine 1-phosphate (S1P) is known to play a role in cellular changes such as differentiation, proliferation, migration, contraction, and survival [6,7,8,9,10,11]. Sphingosine 1-phosphate sphingolipid derivatives are known to have anti-inflammatory, anti-apoptotic, and anti-oxidant activities, and have significant roles in the cardiovascular system [12,13]. Sphingosine 1-phosphate increases the survival of cardiomyocytes during episodes of hypoxia [14]. Some in vitro studies have shown associated decreases in the size of the infarcted area in isolated hearts in both ex vivo and in vivo models [15,16,17]. The role of sphingosine 1-phosphate in cytoprotection through the activation of survival pathways is under intense investigation. Pharmacological post-conditioning with sphingosine 1-phosphate activation has been investigated for potential application after resuscitation in in vitro and in vivo studies [18]. Fingolimod, a sphingosine 1-phosphate receptor agonist, has not been thoroughly investigated in its role as a pharmacological cardioprotective agent for post-cardiac arrest resuscitation care. The present study hypothesis was that fingolimod plays a cardioprotective role in global ischemia–reperfusion injury related to sudden cardiac arrest. Therefore, the aim of this study was to assess the cardioprotective effect of fingolimod through sphingosine 1-phosphate receptor activation after resuscitation from cardiac arrest during ECLS in a rat model. For a better understanding of the cardioprotective mechanism involved, we investigated functional hemodynamic parameters, some of the inflammatory mediators and cardiac biomarkers, Akt1/2 and Erk1/2 pathways, nitro-oxidative stress, apoptosis, and myocardial fibrosis.

## 2. Methods

### 2.1. Study Design and Setting

This study was conducted in the Division of Cardiac Surgery and Translational Surgery Lab, University of Verona Medical School, Verona, Italy.

### 2.2. Animals

Sprague–Dawley rats (300–350 g) were obtained from Harlan Laboratories (Udine, Italy). Rats placed at a density of 2 per cage received the standard diet (rat chow) and had free access to water and they were maintained on a 12 h light/dark cycle at 21 °C. The study was approved by The Ethical Committee of the University of Verona and the National Animal Welfare Committee (BB-CCH#13371, 16/03/2016).

### 2.3. Experimental Design

Rats were orotracheally intubated with an atraumatic tube constituting a venous cannula of 14 G. The rats were then ventilated with a mechanical respirator for rodents (Inhale, Harvard Apparatus, Holliston, MA, USA) with a mixture of oxygen and anesthetic Sevorane 2% (Abbott Laboratories, Sittingbourne, UK), which guaranteed anesthesia for the duration of the procedure, with a fraction of inspired oxygen (FiO_2_) of 90%, a tidal volume of 10 mL/kg; a frequency of 70 breaths per minute of vecuronium bromide (0.1 mg/kg body weight) was administered to obtain complete muscular relaxation and repeated if needed; 1 mg Ketoprofene was administered subcutaneously for analgesia.

Rats were placed in supine position. Thoracic area, ventral surface of the neck, and the hind legs were shaved and skin was disinfected with povidone iodine. A thermocouple microprobe was inserted into the rectum in order to monitor animal temperature during experiment.

Right femoral artery was isolated and a miniaturized catheter with a diameter of 2 Fr (model SPR 838, Millar Instruments, Houston, TX, USA) was inserted for continuous monitoring of systemic blood pressure. Subsequently, the femoral vein was cannulated with a 24 G cannula followed by administration of 500 UI/kg heparin to ensure patency and readiness for ECLS.

Access to heart was achieved via a median sternotomy, and the chest was kept open with a retractor. A venous cannula (a modified four-hole catheter, caliber of 5 Fr) was advanced through the external jugular vein to the right atrium. The left common carotid artery was cannulated with a 24 G catheter which was advanced to the aortic arch and connected to the line of arterial perfusion circuit.

The extracorporeal circulation circuit constituted a roller pump (Stockert SIII, Sorin, Germany), a hollow fiber oxygenator (Sorin, Mirandola, Italy), and a venous reservoir connected to a vacuum to facilitate venous drainage. All were connected by plastic tubing with inner diameters of 1.6 mm. The total fill volume of the extracorporeal circuit including the oxygenator was 6 mL and constituted colloid solution and lactated Ringer’s. The exchange surface of the gas was 450 cm^2^ and the heat exchange surface area was 15.8 cm^2^. Once venous and arterial accesses were in place, cardiac arrest was induced by ventricular fibrillation using a fibrillator with a current of 3.5 mA at 60 Hz released at the level of right ventricular epicardial region. During cardiac arrest, the ventilation was stopped. After 10 min of cardiac and respiratory arrest, ECLS was started and maintained with a flow rate of 80–100mL/kg/min and a mean arterial pressure range of 70–90 mmHg. During ECLS, internal temperature was kept at 35–36°C in both the groups. After 10–15 min of ECLS, spontaneous circulation was restored, ECLS was maintained for 60 min, and then the rats were weaned from extracorporeal circulation.

At the initiation of ECLS, rats were randomly categorized into two groups (Groups A and B). Group A was treated with normal saline, while in animals of Group B, fingolimod was administered at a dose of 1 mg/kg through femoral vein.

**Figure 1:**
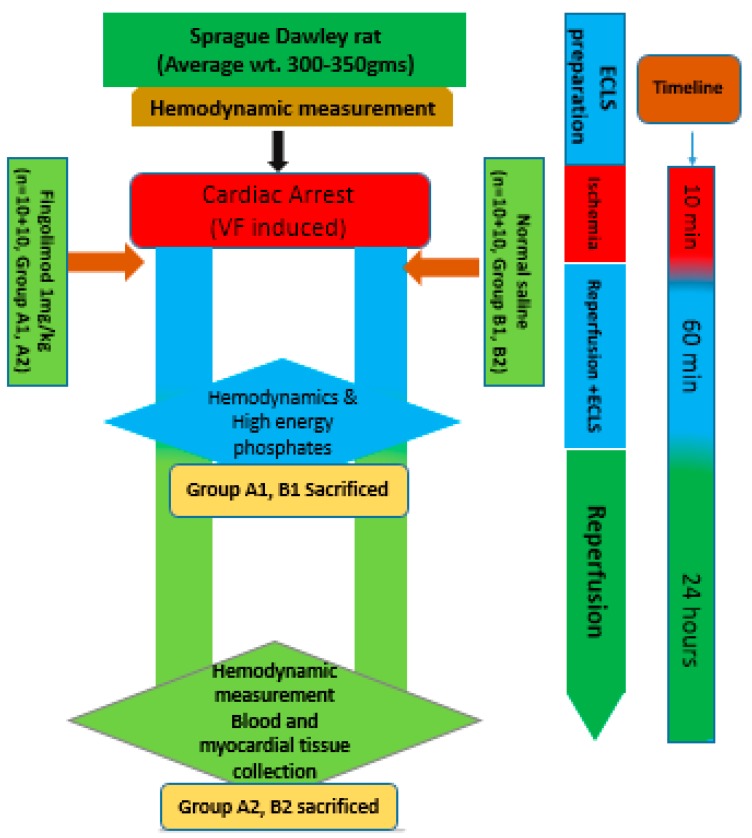
Schematic presentation of experimental study.

In Group A1 and B1, after 1 h of reperfusion, hemodynamic measurements were collected through the Millar catheter inserted into the left ventricle, and myocardial tissue was collected to analyze high energy phosphates and other biomarkers. In the Groups A2 and B2, reperfusion was continued up to 24 h. After 24 h of reperfusion, hemodynamics was measured similarly at 1 h and blood and myocardial tissue were collected for further analysis of inflammation, apoptosis and, oxidative stress. A schematic diagram illustrates the various steps of the experiment (Figure 1).

### 2.4. Hemodynamic Analysis

Determination of hemodynamic parameters was carried out at baseline and after ECLS weaning using a pressure–volume (P-V) conductance catheter with a 2 Fr microtip (SPR-838, Millar Instruments, Houston, TX, USA) which was inserted into the right carotid artery, extending into the left ventricle. Recording of signals was done at a sampling rate of 1000 samples/s using a P-V conductance system (MPVS-400, Millar Instruments). These were stored and displayed on a personal computer by the PowerLab Chart 5 Software System (AD Instruments, Colorado Springs, CO, USA). Hemodynamic parameters were determined by recording left ventricular end systolic and diastolic pressures along with contractility index (dp/dt min and dp/dt max).

### 2.5. Analysis of Serum Inflammatory Mediators and Biomarkers

After the ischemia and reperfusion phase, blood was collected and placed at room temperature for 30 min. Serum was collected and the concentrations of serum inflammatory mediators TNF-α, IL-6, ICAM-1, and IL-1β were determined using ELISA-based kits obtained from Thermo Scientific (Rockford, IL, USA). The serum levels of CK-MB and cardiac Troponin-I were measured using CK-MB and Cardiac Troponin-I Assay Kits (Sigma-Aldrich, Gillingham, UK).

### 2.6. Quantification of Oxidative Stress

For determination of malondialdehyde (MDA) and reactive oxygen species (ROS) in myocardial tissue, a commercially available method was adopted (Thermo fisher, Waltham, MA, USA); tissue pieces were processed at 37 °C, 5% CO_2_ 95% air atmosphere for 60 min according to the manufacturer’s instructions. The myocardial tissue was incubated at 37 °C for 30 min in PBS (137 mM NaCl, 2.7 mM KCl, 9.8 mM Na_2_HPO_4_, 1.5 mM KH_2_PO_4_, pH 7.3). Absorbance at 490 nm was measured with a microplate reader (EL × 800; Bio-Tek, Winooski, VT, USA).

For determination of nitrogen reactive oxygen species (peroxynitrites), the nitrosylation of cardiac proteins was measured using an antibody against nitrotyrosine, as described in the following section.

### 2.7. Immunoblotting Analysis

For immunoblotting assays, rabbit antibodies for non-phosphospecific Akt1/2 and ERK1/2 goat anti-rabbit IgG conjugated with horseradish peroxidase (HRP) were purchased from Abcam (Cambridge, UK), while rabbit anti-phosphorylated Akt1/2 and ERK1/2 antibodies were purchased from (Cell Signaling, Beverly, MA, USA). The remaining reagents were purchased from (Sigma Chemical, St. Louis, MO, USA).

Homogenization of heart tissue was carried out in a buffer solution containing 1% Triton 100-X and phosphatase and protease inhibitors cocktail (Sigma Chemical, St. Louis, MO, USA) as described by Giani et al. [19]. After centrifugation of the homogenate at 16,000 rpm for 15 min at 4 °C, the supernatant was collected in separate aliquots. Protein concentration of the tissue extract was measured using the BCA assay kit (Beyotime Institute of Biotechnology, Jiangsu, China). For analysis of phosphorylation levels of Akt1/2 and ERK1/2, equal amounts of solubilized proteins (35 μg) were denatured by boiling for 5 min at 100 °C in reducing buffer, and then resolved by SDS-PAGE. The protein was then transferred to polyvinylidene difluoride (PVDF) membranes. PVDF membranes were pre-wetted with methanol and distilled water prior to submersion in 30% methanol containing transfer buffer. After transfer of protein, the membranes were incubated for 1 h in blocking solution that contained 5% FBS in TBS containing 0.1% Tween 20. After blocking, primary antibodies were diluted (1:1000) in blocking solution and incubated over night at 4 °C with unphosphorylated and phosphospecific antibodies. Equal loading of protein in the acrylamide gels was confirmed by reusing the PVDF membranes with antibody against an anti-GADPH. Unbound primary antibody was washed extensively from the membranes with TBS-T solution for 20 min. These membranes were then incubated with secondary antibodies conjugated with HRP at a rate dilution of (1:10,000) and substrate of HRP chemiluminescence as a result of reaction was measured through a Syngene Western blotting detection system. Protein band densities were analyzed using ImageJ 1.37 software.

### 2.8. Immunohistochemical Staining

Heart tissue sections of 3 um thickness were placed on slides and heated to 60 °C for 1 h, and then rehydrated using graded concentration ethanol. Antigen retrieval was carried out using appropriate buffer and the procedure explained previously [12]. Endogenous peroxidase activity was blocked by incubation of tissue section, carried out at 4 °C overnight. This was followed by washing of primary antibodies and incubation with biotin-conjugated secondary antibody for 30 min and di-aminobenzidine and hydrogen peroxide chromogen substrate (Dako Corp., Carpinteria, CA, USA). Counter staining of all slides was done with hematoxylin. Rabbit IgG in place of primary antibody was used as a negative control. Slides were examined using a Nikon E400 microscope (Nikon Instrument Group, Melville, NY, USA).

### 2.9. High-Energy Phosphates

High-performance liquid chromatography was used to quantify adenosine monophosphate (AMP), adenosine diphosphate (ADP), adenosine triphosphate (ATP), and phosphocreatine (PCr), as elaborated in a previous publication [20]. Calculation of energy charge (EC) was done according to the following formula: EC = (ATP + ½ ADP)/(ATP + ADP + AMP) [21].

### 2.10. TUNEL Assay

Heart tissue was deparaffinized with 100% xylene following serial concentrations of ethanol. Apoptotic cells were stained and the Click-it plus terminal deoxynucleotidyl transferase dUTP nick end labeling (TUNEL) assay was used for the in situ apoptosis assay. (Molecular Probes Life Technology, Thermo Scientific). Hoechst 33342 staining solution was used for examining nuclei [12].

### 2.11. Histology and Interstitial Fibrosis Determination

Three micron sections obtained from the paraffin-embedded left ventricular myocardial tissue were stained with hematoxylin–eosin staining, and histological measurements were expressed as a diameter (μm) of cardiomyocyte. Masson’s trichrome was used to evaluate cardiac fibrosis as mentioned in previously [22].

### 2.12. Statistical Analysis

All measurements and results are presented as mean ± SD. Control and treatment groups were compared using Student’s t-test or Mann–Whitney U nonparametric test. Analyses were performed using SPSS software version 21 (SPSS Inc., Chicago, IL, USA) and a *p*-value < 0.05 was considered significant.

## 3. Results

### 3.1. Left Ventricular Function

Regarding baseline hemodynamic parameters, no significant difference was seen between control and treatment groups (Table 1). All rats were subjected global ischemia through CA following fingolimod treatment in Groups B1 and B2 and saline treatment in Group A1 and A2 (*n* = 15 in each group). Hemodynamic parameter measurements were done to evaluate LV performance using the Millar catheter system.

**Figure 2:**
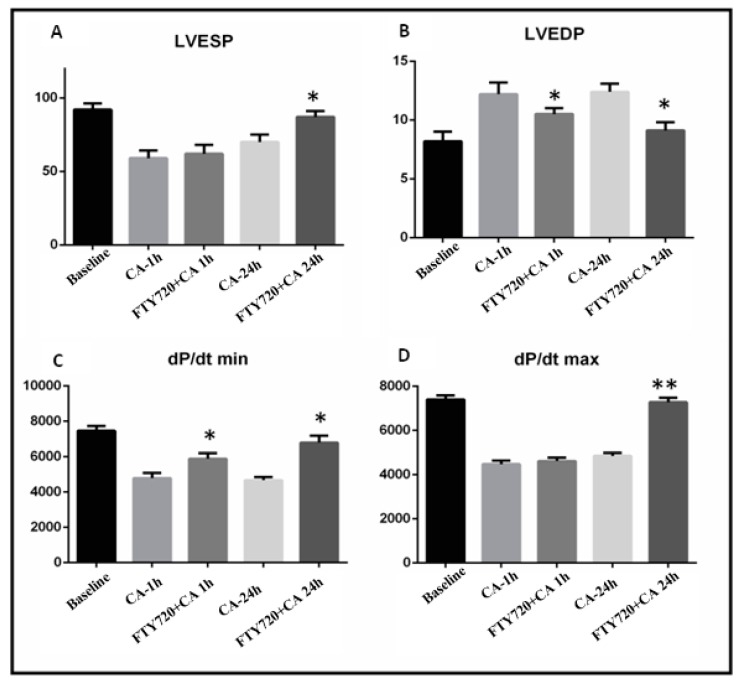
Hemodynamic parameters measured at baseline after 1 h and 24 h of reperfusion. Effects of FTY720 on left ventricular function in the rats with a CA-reperfusion-induced injury. Following 10 min of CA, treatment was administered at the start of reperfusion. (**A**) Effects of FTY720 on LVESP. (**B**) Effects of FTY720 on LVEDP. (**C**) Effects of FTY720 on LV dp/dt min. (**D**) Effects of FTY720 on LV dP/dt max. LVESP and LVEDP were measured using a multichannel physiological recorder. LV dP/dt max and min are expressed as mmHg/sec. LVESP and LVEDP are expressed as mmHg. LV dP/dt max: the rate of maximum positive left ventricular pressure development; LV dP/dt max: the rate of maximum negative left ventricular pressure development; LVESP: left ventricular end-systolic pressure; LVEDP: left ventricular end-diastolic pressure. Values are expressed as means ± SD. * *p* < 0.05, ** *p* < 0.001.

The LV end systolic pressure (LVESP) elevated significantly after CA and resuscitation in Group B2 as compared to Group A2 (*p* ≤ 0.05) (Figure 2A). No difference between Groups A1 and B1 was observed (*p* > 0.05). The LV end diastolic pressure (LVEDP) measurements showed a reduction in Groups B1 and B2 vs. Groups A1 and A2 (*p* ≤ 0.05) respectively (Figure 2B). The minimal pressure relaxation rate (dP/dt min) was also found to be improved in Groups B and D as compared to Group A1 and Group A2 (*p* ≤ 0.05) (Figure 2C). Ventricular systolic performance dp/dt max after CA and reperfusion was increased in Group B2 vs. Group A2 (*p* ≤ 0.05) (Figure 2D).

### 3.2. Serum Levels of Inflammatory Mediators and Cardiac Markers

**Figure 3:**
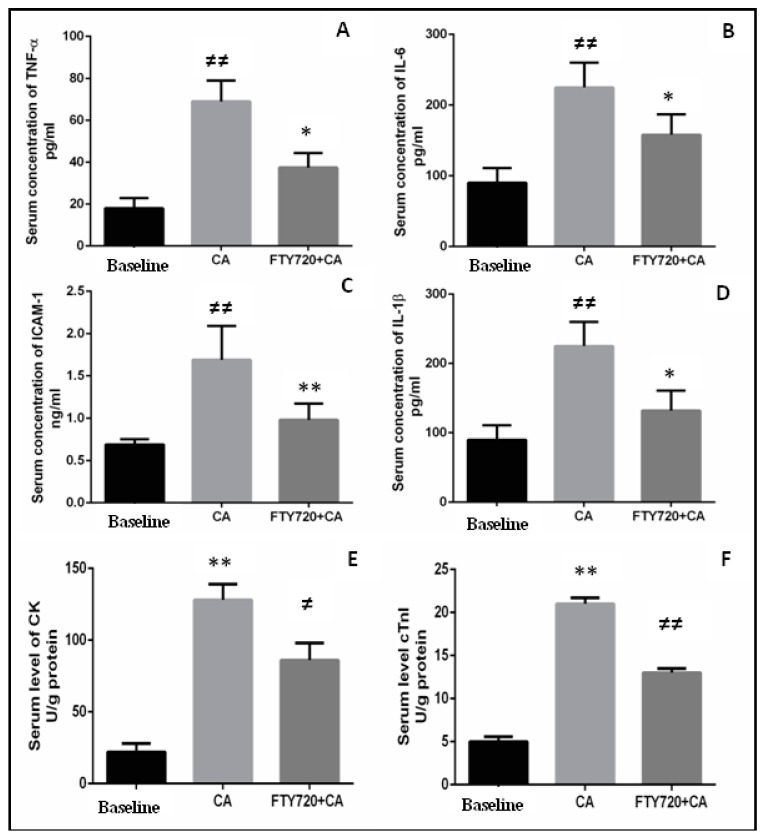
Myocardial production of TNF-α, IL-6, ICAM-1, and IL-1β after 10 min CA and 24 h of reperfusion. (**A**) CA model without fingolimod treatment showed high expression of TNF-α compared to the model with fingolimod treatment. (**B**) CA-reperfusion induced significantly heightened IL-6 after 24 h of reperfusion compared with the fingolimod-treated and baseline groups. (**C**) FTY720-treated group showed a remarkably reduced production of ICAM-1 as compared to control. (**D**) Production of 1L-1β was higher in control vs. FTY720-treated group in CA–reperfusion group. (**E**,**F**) Serum levels of creatinine kinase-MB (CK-MB) and cardiac Troponin I (cTnI) in the serum of rats in the baseline, CA-reperfusion, and CA-reperfusion + fingolimod group (1 mg/kg) groups. Each bar height represents the mean ± SD (each group *n* = 15). (^##^ *p* ≤ 0.01 vs. baseline. * *p* ≤ 0.05 and ** *p* ≤ 0.01 vs. CA control group).

In comparison with the baseline, CA-related I/R injury was marked by elevation in the levels of cytokines, mainly TNF-α, IL-1β, IL-6, and ICAM-1. All these cytokines were increased in serum in response to ischemia and reperfusion. In the present study, these mediators were measured in CA–reperfusion-related I/R injury. Although, ICAM-1 was independently increased in response to I/R, in CA-related-ischemia–reperfusion injury, elevation was observed. Compared to baseline serum levels of TNF-α, IL-6, IL-1β, and ICAM-1, serum levels were significantly increased in CA–reperfusion group (*p* ≤ 0.001). On administration of fingolimod (1 mg/kg) there was attenuation in serum levels of TNF-α, IL-6, 1L-1β, and ICAM-1 compared to the control group (*p* ≤ 0.05), *p* ≤ 0.05, *p* ≤ 0.05, and *p* ≤ 0.001) respectively), as shown in Figure 3A–D.

Cardiac markers of cardiomyocytes injury, the serum levels of CK-MB and cTnI, were found to be 22 ± 9.3 and 5.35 ± 0.60 U/g protein in the baseline-operated group. These were significantly increased in the CA–reperfusion group to 128.8 ± 10.9 and 21.72 ± 0.99 U/g protein, respectively. After treatment with fingolimod, the CK-MB and cTnI levels were significantly decreased (Figure 3E,F).

### 3.3. Effect of Fingolimod on Nitro-Oxidative Stress

**Figure 4:**
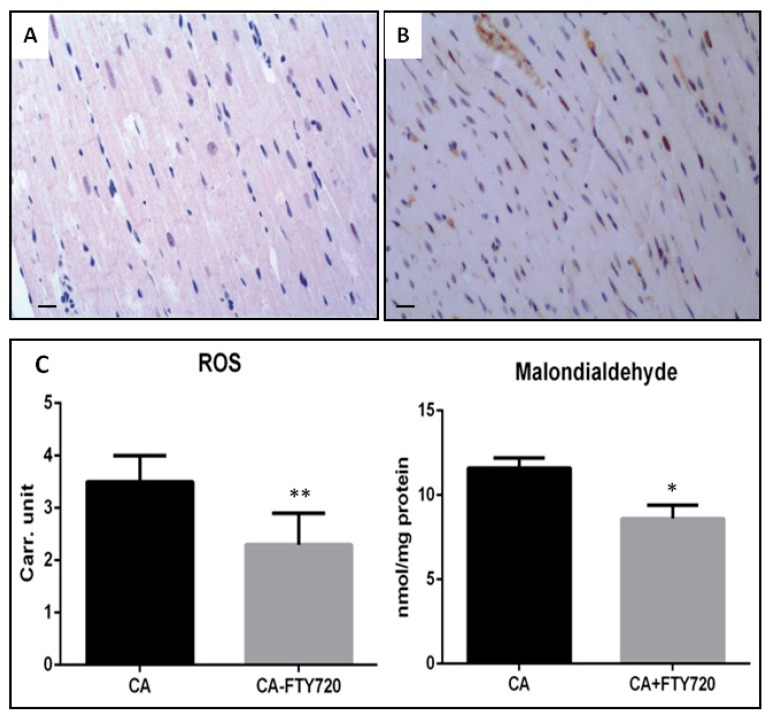
Myocardial nitrotyrosine staining of (**A**) FTY720-treated group (*n* = 15 each) and (**B**) Control group (*n* = 15 each), Rats were subjected to 10 min CA followed by 24 h reperfusion. At the end of the experiment, the heart was removed, and nitrotyrosine localization was determined. Magnification 20×, scale bar 100 μm. (**C**) Comparison of oxidative stress in fingolimod-treated and control group. ROS: reactive oxygen species, CA: cardiac arrest, FTY720: fingolimod; Carr. unit: Carratelli unit. (** *p* ≤ 0.001, * *p* ≤ 0.05).

Nitrative stress plays an important role in myocardial tissue injury under ischemia–reperfusion. Excessive NO production from NOS and the reaction product between NO and peroxynitrite superoxide has been observed to activate apoptotic signaling pathways, leading to apoptotic cell death. To examine whether fingolimod reduced myocardial nitrative stress caused by CA-related ischemia–reperfusion, and thus attenuation in myocardial apoptosis, we determined peroxynitrite expression. As illustrated in Figure 4 A, B, fingolimod markedly downregulated peroxynitrite expression. This was shown by indicates decreased production of nitrotyrosine.

To examine whether fingolimod regulates free radical production in ischemia–reperfusion, we examined free oxygen radicals and aldehydes (lipid peroxidation derivatives) in the frozen samples of myocardial tissue. In the CA control group, reactive oxygen species levels were found to be higher as compared to those in the CA–fingolimod-treated group, as determined by chromatography and mass spectrometry. Overall, our results showed that the fingolimod treatment in CA decreases oxidative stress (Figure 4C).

### 3.4. Bcl-2 and Bax Signaling Pathways

**Figure 5:**
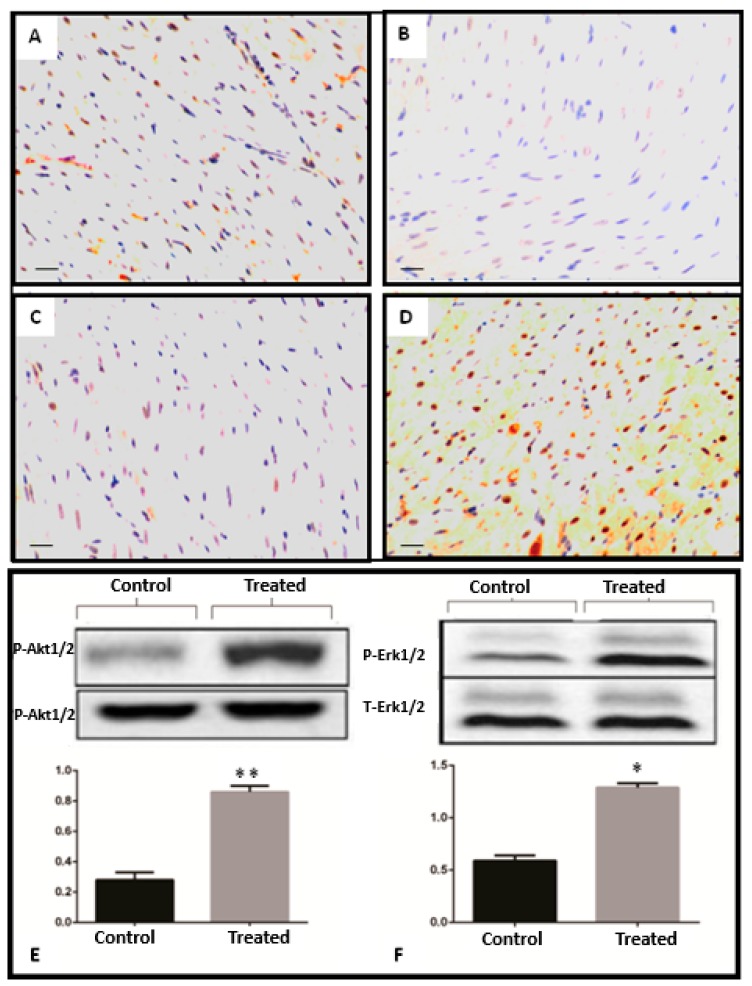
Myocardial tissue expression levels of Bcl-2 and Bax after 10 min of CA and 24 h reperfusion. The protein expression levels of Bcl-2 and BAX were determined by immunohistochemistry, (**A**) BAX expression in CA control group, (**B**) Bax expression in CA–FTY720-treated group (control vs. treated *p* ≤ 0.05). (**C**) Bcl-2 expression in control CA group and (**D**) Bcl-2 expression in FTY720-treated group (control vs. treated (*p* ≤ 0.05)). Magnification 20×, scale bar 100 μm. Representative western blot and relative density ratio of the phosphorylated (*p*) form of Akt1/2 (D) ERK1/2; (**E**) samples of left ventricle at the end of 24 h reperfusion. Relative densities showed that fingolimod activates phosphorylation of these proteins. Values presented are means ± SD; (*n* =15 samples/group). (* *p* ≤ 0.05, ** *p* ≤ 0.001).

After 10 min of CA and 24 h of reperfusion, myocardial tissue was excised for immunohistological staining to measure the expression levels of anti-apoptotic (Bcl-2) and pro-apoptotic (Bax) proteins. In the fingolimod-treated group (Group B2), Bcl-2 expression was significantly increased versus the CA control group (Group A2), while expression of Bax was down-regulated in Group B2 as compared to Group A2 (*p* ≤ 0.05). This indicates that fingolimod during CA decreases apoptosis by upregulating Bcl-2 and downregulating Bax proteins (Figure 5A–D).

### 3.5. Effect of Fingolimod on Erk1/2 and Akt1/2 Signaling Pathways

Pro-survival signaling pathways were measured in the myocardial tissue. The Akt/PI3 kinase pathway is a signaling pathway important for survival. We measured the phosphorylation level of Akt1/2 in Groups A2 and B2 in the myocardial tissue. As shown in Figure 3E, phosphorylation levels of Akt1/2 were higher compared to the control group (*p* ≤ 0.05). ERK1/2 phosphorylation was also analyzed by western blot in rat myocardial samples. As shown in Figure 3F, phosphorylation level of ERK1/2 was found to be increased in Group B2 compared to Group A2 (*p* ≤ 0.05) (Figure 3E,F).

### 3.6. High-Energy Phosphates

**Figure 6:**
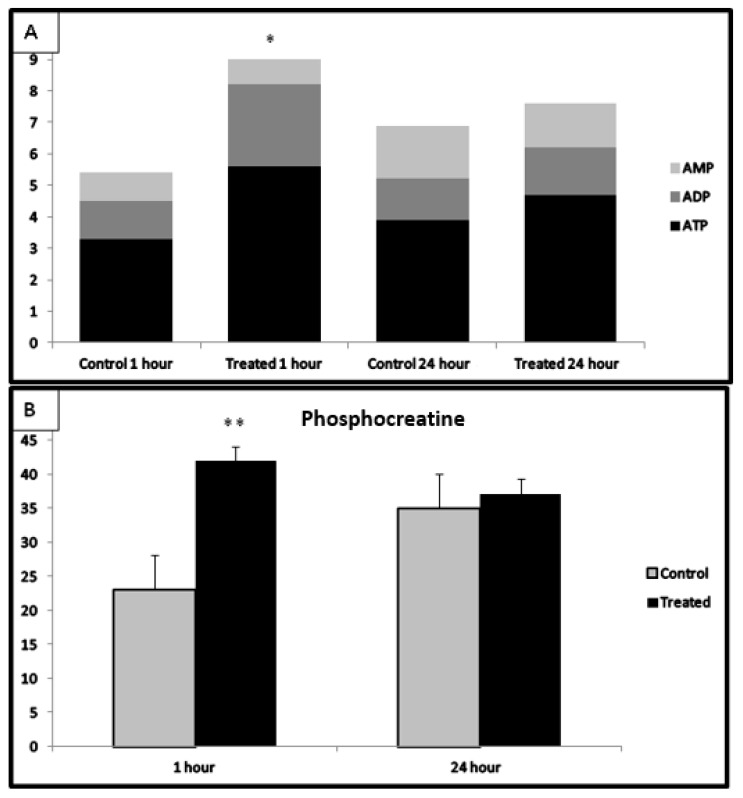
High-energy phosphates in myocardial tissue of the LV in the fingolimod-treated groups compared with the control group. (**A**) AMP, ADP, and ATP levels at 1 h and 24 h of reperfusion; (**B**) changes in phosphocreatine, (* *p* ≤ 0.05, ** *p* ≤ 0.001). AMP: adenosine monophosphate; ADP, adenosine diphosphate; ATP, adenosine triphosphate.

In the model of CA and resuscitation, measurement of high energy phosphates revealed superior preservation in myocardium following fingolimod treatment (Figure 6). Phosphocreatine was significantly higher in the fingolimod-treated group of animals compared with the control group tissue (*p* ≤ 0.001), whereas ATP was significantly increased after both the 1 h and 24 h of reperfusion in treated group compared to the control group (*p* ≤ 0.05). Energy charge was significantly elevated after 1 h of reperfusion in the fingolimod-treated group as compared with the control group (0.85 ± 0.12 vs. 0.63 ± 0.15; *p* ≤ 0.05). Even after 24 h reperfusion, it remained significantly higher compared to the control group (0.83 ± 0.13).

### 3.7. Effect of Fingolimod on Apoptosis

**Figure 7:**
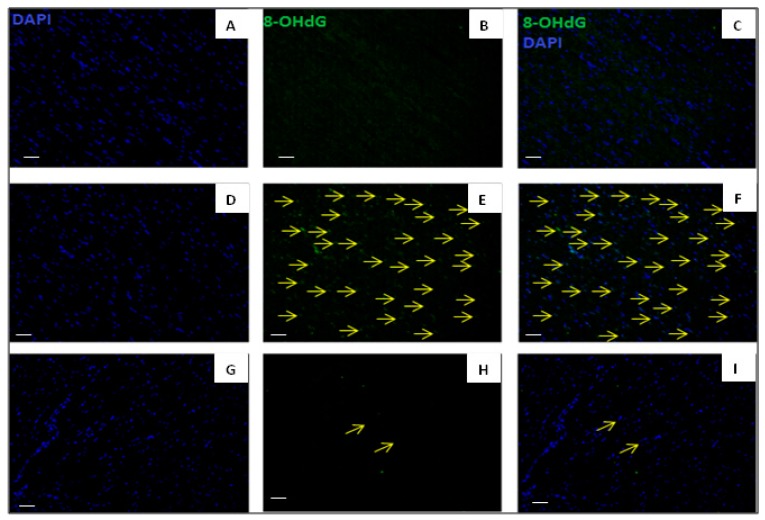
Representative photomicrographs of immunofluorescent staining for TUNEL-positive nuclei in the baseline, I/R control, and I/R–fingolimod groups. (**A**,**D**,**G**) DAPI in myocardial tissue, (**B**,**E**,**H**) positive TUNEL signals, and (**C**,**F**,**I**) merged images. TUNEL-positive myocytes were much lower in numbers frequently in control CA group than in CA-FTY720 group. Original magnification 20×. Scale bar 100 μm.

The TUNEL assay is a standard way to assess apoptosis. Group A2 showed extensive apoptosis. However, S1P receptor activation by fingolimod presented a significant attenuation of TUNEL-positive nuclei in myocardial tissue following cardiac arrest and reperfusion. This indicates that fingolimod exerts an anti-apoptotic role in I/R related to CA (Figure 7).

### 3.8. Effect of Fingolimod on Collagen Deposition and Neutrophil Infiltration

**Figure 8:**
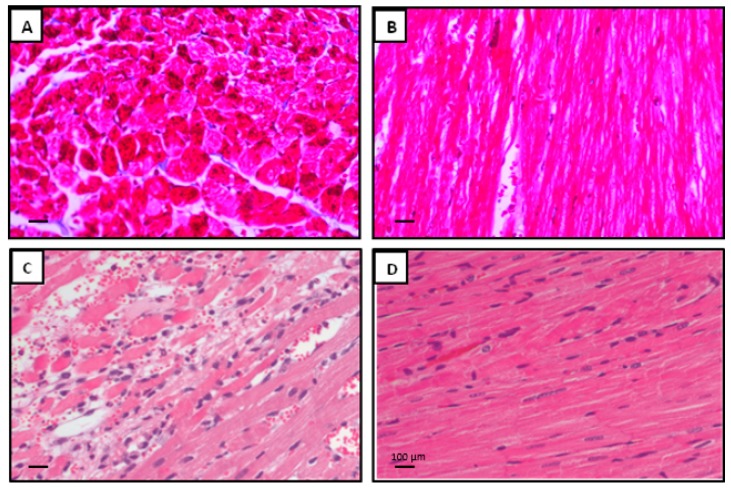
(**A**,**B**) Representative photomicrograph showing the Masson’s trichome stain, illustrating collagen deposition in CA-induced ischemia–reperfusion myocardium after 24 h. FTY720 was attenuated following CA myocardial interstitial collagen deposition. The viable myocardium is stained bright red. Collagen fibers are stained bright blue. (**C**,**D**) Representative photomicrograph showing the histopathological changes in CA-induced ischemia–reperfusion: (**C**) cardiac section showing interstitial edema and neutrophil infiltration in control and (**D**) heart tissue section showing mild necrosis and band contractions in fingolimod-treated group (20 × magnification).

Interstitial collagen deposition was measured by collagen volume fraction (CVF) to evaluate myocardial fibrosis after VF-induced CA and reperfusion for 24 h. The interstitial collagen deposition in the late phase of reperfusion was significantly reduced in the fingolimod-treated group (Figure 8A,B). To calculate CVF, we measured the collagen staining expression (blue) in Masson’s trichrome-stained images. In CA-I/R group, we observed interstitial edema and structural disarray, including neutrophil infiltration. However, pre-ischemia fingolimod treatment reduced morphological changes and neutrophil infiltration remarkably. The difference between control and treated group was statistically significant (*p* ≤ 0.05) (Figure 8C,D).

## 4. Discussion

Sudden cardiac arrest is a major health concern worldwide, and is the most frequent cause of death. Various cardioprotective strategies to prevent acute global ischemia–reperfusion injury has been tested. In the last two decades, multiple experimental studies have been carried out to identify the cardioprotective roles of various substances including volatile anesthetic agents [23,24,25], sodium hydrogen exchange inhibitors [26], statins [27], pharmacological preconditioning agents [28], and anti-inflammatory drugs [29]. A vast majority of these preclinical strategies could not work in actual clinical settings. In the present study, administration of fingolimod resulted in decreased inflammation, oxidative stress, and preservation of high energy phosphates in cardiomyocytes, which may contribute to enhanced cardioprotection. Our results showed inhibition of pro-apoptotic and activation of anti-apoptotic proteins, and reduction in oxidative and nitrative stress. Our results agree with the earlier findings that the phosphorylation of Akt1/2 and Erk1/2 is important for cell survival pathways [30,31,32].

Several studies have suggested a cardioprotective role of fingolimod through activation of survival pathways in rodent models [15,16]. One such study used S1P receptor knock-out mice showed a high level of myocardial damage as compared to wild-type mice [33]. In another study, mice lacking in sphingosine kinases were found to have greater size of infarction as compared to control mice when subjected to cardiac arrest. Studies have shown that not only the activation of S1P receptors, but also the metabolism of S1P is important in pre- and post-conditioning cardioprotective mechanisms [34,35].

Ischemia–reperfusion injury can be caused by different mechanisms and pathways. Activation of apoptotic pathways, complement system activation, increased inflammation, and oxidative stress can cause myocardial injury following ischemia–reperfusion [36]. Fingolimod shows potential to deal with most of the above mentioned myocardial damaging mechanisms to prevent I/R injury. Reperfusion after transient ischemia in myocardium leads to apoptosis in cardiomyocytes and cardiac dysfunction [37,38]. TUNEL-positive nuclei staining, i.e., the standard method to measure level of apoptosis, was used in this study. Our investigation was consistent with previous studies [12] showing that rats treated with fingolimod expressed a lower frequency of TUNEL-positive nuclei after 24 h of reperfusion, supporting the cardioprotective role of fingolimod by activating anti-apoptotic cascade.

S1P receptor agonists have important roles in immune suppression [39]. A porcine model of I/R [17] and a spontaneous obstructive coronary atherosclerosis murine model [40] tested for immunosuppression, showed reduction in infarct size and mortality in the fingolimod-treated group. In the present study, a significantly lower concentration of pro-inflammatory cytokines was found in blood and tissues subjected to inflammation, a finding concordant with the previous data [40].

ICAM-1, IL-6, IL-1β, and TNF-α contribute to develop inflammatory mechanisms as pro-inflammatory cytokines [41,42,43]. A correlation was found between the anti-inflammatory effect of fingolimod on cardioprotection. I/R increased ICAM-1, IL-6, IL-1β, and TNF-α levels in the control group, whereas fingolimod treatment decreased the concentrations of these cytokines. Therefore, the suppression of inflammatory cytokines by fingolimod treatment may protect the myocardium from I/R injury caused by pro-inflammatory cytokines, as evident from the present study.

One of the main targets of this drug was to mitigate apoptosis in I/R. During I/R, molecular signaling via RISK and SAFE pathway activation has been reported [16,44]. The RISK (Akt1/2, Erk1/2, and GSK-3β) and SAFE (JAK and STAT3) pathways are main sources for mitigation of apoptosis by preventing opening of the mitochondrial permeability transition pore [45,46,47]. Consistent with the previous findings, activation of RISK and SAFE signaling pathways was observed following decreased level of apoptosis in treated group compared to the control. The inhibition of pro-apoptotic proteins Bax and enhanced immunoreaction for anti-apoptotic protein Bcl-2 was found after 10 min of CA and 24 h of reperfusion.

This study was not without limitations. Sudden cardiac arrest patients may have other underlying pathologies and co-morbidities including diabetes, hypertension, and hyperlipidemia. Our experimental model did not truly reflect those conditions; this could be the reason that many studies on experimental models have failed to show the same beneficial effects in actual clinical practice. However, in a recent study, we showed that the rat model, due to similarities in the SIP receptors between rat and human heart, is a more appropriate model to study the beneficial effects of fingolimod in clinical trials [13].

In our study, maximum reperfusion time was 24 h, and we did not study the effect of fingolimod in late phase. Prolonged reperfusion could have provided more strong evidence of reduction in I/R injury. The effect of fingolimod in structural remodeling can be studied only if there is prolonged reperfusion time. However, due to complex surgical procedures, it was hard to achieve this in our model.

Furthermore, we administered only one dose before initiating ischemia in our model. There is a possibility that multiple doses of fingolimod could offer better cardioprotection. Further studies need to be conducted in order to check the efficacy of multiple doses. 

Although fingolimod is generally considered safe and well tolerated, adverse effects of transient bradycardia and atrioventricular block have been reported nonetheless. These adverse effects were resolved spontaneously in majority of the cases. Moreover, it is recommended to use fingolimod in hospital settings under strict monitoring so that the likelihood of these adverse effects can be minimized using timely counter medication if required.

In spite of these limitations, we speculate that fingolimod has great potential to be used for cardioprotection in cardiac arrest. On the basis of these findings, it can be suggested that sphingosine 1-phosphate receptor activation in patients with cardiac arrest could be most important for assessment of the potential clinical efficacy of this cardioprotective agent.

## 5. Conclusions

In conclusion, fingolimod plays important role in preservation of cardiac mechanical functions, reduction in myocardial apoptosis, inflammation, and nitro-oxidative stress. The potential cardioprotective mechanism is associated with activation of the reperfusion injury salvage kinase and survivor activating factor enhancement pathways. Furthermore, hemodynamic parameters improved in the late phase of reperfusion. The results of this study might be a potential source for translation into clinical treatment.

## Figures and Tables

**Table 1:** Comparison of baseline characteristics among different groups.

Baseline	Group A1	Group B1	*p* Value	Group A2	Group B2	*p*-Value
HR (beats/min)	281 ± 16	278 ± 21	Ns	309 ± 29	295 ± 24	ns
MAP (mmHg)	137 ± 16	132 ± 18	Ns	112 ± 14	116 ± 16	ns
CO (mL/min)	42 ± 3.5	45 ± 4.2	Ns	44 ± 3	47 ± 5.2	ns
